# Integrated information for integrated care in the general practice setting in Italy: using social network analysis to go beyond the diagnosis of frailty in the elderly

**DOI:** 10.1186/s40169-016-0105-6

**Published:** 2016-07-27

**Authors:** Michela Franchini, Stefania Pieroni, Loredana Fortunato, Tamara Knezevic, Michael Liebman, Sabrina Molinaro

**Affiliations:** 1 Institute of Clinical Physiology, Italian National Research Council–CNR, Via Moruzzi, 1, 56124 Pisa, Italy; 2 IPQ Analytics, LLC/Strategic Medicine, Philadelphia, Pennsylvania USA

**Keywords:** Frailty, Elderly, General practice, Population-based planning, Data linkage, Social network analysis, Disease trajectory

## Abstract

**Background:**

Frailty has been defined in different ways and several diagnostic tools exist, but most of them are not applicable in routine primary care. Nonetheless, general practitioners (GPs) have a natural advantage in identifying frailty, due to their continued access to patients, patient-centered approach and training. GPs have also an advantage in conducting population-based evaluation as consequence of their role of gatekeepers of the health care system. This paper aims to identify those socio-demographic and clinical profiles and the relative information sources that, from the GPs’ perspective, act as frailty markers, not solely as a diagnosis of state but as the ability to identify a patient’s trajectory, over time, through the aging process.

**Methods:**

This study was performed as a survey within a population aged 75 and over, attending 148 GPs in Italy. A total of 23,996 patients were classified by GPs in distinct frailty status, without the use of a specific evaluation tool, but only referring to general indications. Co-morbidity was objectively assessed by a record-linkage with previous hospitalizations, in order to assess the occurrence of previous illnesses that could be associated with the likelihood of being identified as frails or at risk. The methodological approach is based on social network analysis (SNA), suited to explore relational aspects of complex phenomena.

**Results:**

Our findings reveal that GPs are able to perform low cost population-based evaluation, by exploiting the advantages of their approach to patients, combined with the information derived from their daily practice and from other sources currently available.

**Conclusion:**

We believe that informative integration among different sources of available data can provide a comprehensive picture of the health state of patients in a shorter time and at lower cost. The identification of limited patient trajectories based on these observations can enable the development of critical biomarkers/diagnostics and prognostic indicators that will enhance patient care and potentially reduce inappropriate healthcare use. We also believe that network analysis is an extremely flexible research tool and a rich theoretical paradigm, and it may be used in the healthcare planning.

## Background

Globally, the population is aging, i.e. the average age is increasing annually in most countries. This results from two primary causes: (1) improved levels of and access to healthcare; and (2) lowered birth rates. Between 2007 and 2040, the Europe anticipates the population over 60 will increase from 25.7 to 39.9 % (Italy) compared with 17.2 to 25.4 % (US) [1]. The aging population will increase demand for care services for the frail elderly [2] as noted in the US, but the elderly only represented 13 % of the population, they incurred 34 % of all healthcare spending [3]. It is estimated that by 2060 the disabled elderly in Europe who need care will increase by 115 % and the long term-care public expenditure ratio to the European gross domestic product will grow from 1.2 to 2.5 [4].

Frailty represents the transition for a patient from their presentation of geriatric syndrome (e.g. incontinence, falls, pressure ulcers, delirium and functional decline, etc.) to a state where disability/dependence, nursing home treatment and death are the likely outcomes [5]. Thus proactive identification of early signs of frailty in the elderly and the appropriate planning of their care pathway are essential not only for improving quality of life but also for cost optimization [6].

According to its multidimensional definition, the frailty syndrome results from the complex interaction of multiple factors, e.g. an individual’s level of function [7–10], extent of social isolation and their psychological profile [11–13]. Moreover, frailty results from a dynamic process that extends far beyond biological vulnerability [14] and leads to or is determined by different multi-morbidity patterns, consisting of the co-occurrence of unrelated diseases, i.e. the transition from geriatric syndrome to frailty syndrome.

Identifying frailty requires the evaluation of the complete patient [15], something that may be readily supported by a generalist’s knowledge and their longitudinal observation of the patient [16].

Several authors conclude that a first step in frailty assessment should be done routinely by the general practitioner (GP) [17] using tools integrated into their daily practice [18–21]. Unfortunately, although current literature agrees on the bio-psychosocial model of frailty [17], its operational definition is yet unresolved [10, 11, 13, 22]. Two definitions predominate: the phenotype [7] and the accumulation of deficits [23, 24] but several variants have been proposed [9, 13, 20, 21, 25–27].

Their usability within the primary care setting may, however, be limited [18, 21]. These studies on frailty assessment have focused on establishing an instrument that will yield a diagnosis by typically including non-frailty, pre-frailty and frailty designations. While beneficial in identifying patients at risk, the simple classification may be limited in establishing optimal personalized patient management because they focus on patient state and not the dynamics of the patient condition.

**Fig. 1:**
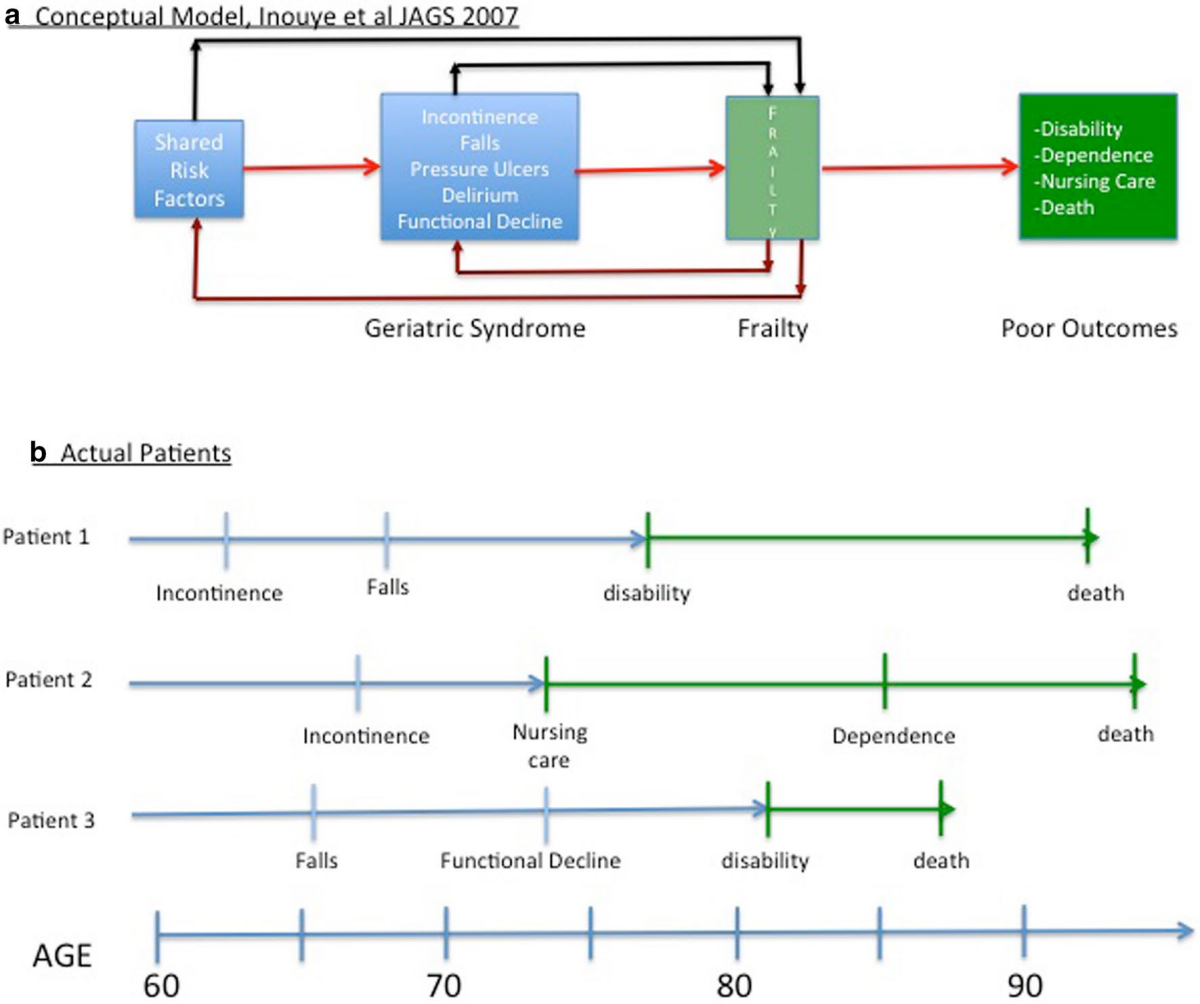
**a** Disease stratification, staging and prognosis in the frailty syndrome; **b** personalized approach based on disease process and patient specificity

It is limiting to classify a patient’s “state”, but this is not unique to frailty and is present in most medical diagnoses. This issue is critical because most diseases are actually processes that evolve over time, as noted above, and not static states. In particular, the elderly patient progresses through two syndromes, the geriatric syndrome and frailty (Fig. 1a) and not two clearly defined diseases [5]. Therefore it can be even more important to understand how to critically evaluate the patient: (1) what is the trajectory of their specific condition (disease stratification); (2) how far along that trajectory have they progressed (disease staging); and (3) how rapidly are they progressing (prognostic velocity). These components can form the basis for improving individualized (personalized) diagnosis and potentially optimizing personalized treatment planning and outcome (Fig. 1b).

GPs require simple instruments based on data available in electronic patient records [18] to facilitate frailty assessment as reliably as possible [21]. The GP’s role, whenever frailty is suspected, involves suggesting that the patient undergo more specific clinical assessment, to identify optimal preventive and healthcare interventions.

In this study we assume that GPs have a natural advantage in the early identification of frail and at-risk elderly patients, due to the special interactions and clinical features of primary care including first contact access, person-focused care over time, preventive approach and information availability [28].

The opportunity exists for the GP to go beyond the diagnosis of frailty, especially with the potential aid of the instruments being developed, but to also identify if there might be patterns of progression that appear in the populations that they treat.

This study focuses on identifying potential trajectories in this transition as defined by sub-populations of patients who present with similar profiles, based on the data generated through the contacts that patients have with the healthcare system. These contacts collectively form a patient’s individual electronic health record.

The application of social network analysis (SNA) to identify such potential sub-groups could enhance identification of key risk factors, and improve both early diagnosis and treatment/management. SNA is well suited to explore relational aspects of complex phenomena, also referring to a set of individual and group metrics. At the level of network metrics, we firstly aim to identify those sub-networks (communities or clusters or fractions) of nodes showing higher levels of linkage density with the frailty levels identified by GPs. Moreover we aim to identify those elderly characteristics which play a central role within each community [29].

We are also interested in understanding if a previous experience of hospitalization for specific clinical features during the last 12 months could have influenced GPs frailty assessment.

Finally we want to show how the network paradigm, based on the strong theoretical foundation of graph theory, could provide important contributions in the areas of public health research and planning.

## Methods

### Social network analysis

The availability of electronic records generated during the interactions among the patient, their GPs and the health care infrastructure poses new challenges to information technology to reduce the gap between data and knowledge.

In this scenario, social networks can present a validated framework to analyze large and heterogeneous data from different sources, reveal the relationships among them and to identify criteria that form “community information” [30]. SNA focuses on the importance of relationships among interacting units. It involves a set of actors (or units or nodes) that have relationships (or linkages or ties) with one another [30]. One of the main themes of the SNA is the way in which individual actors determine social structures by their pattern of interaction and, at the same time, how macro-structure establishes the interaction among individuals.

Data visualization using graphical representation enables various networks to be communicated among individuals and compared across different systems or levels of information.

The descriptive analyses of network properties provide details concerning the node positions, properties of network subgroups or characteristics of the entire network [31, 32].

To determine the structural properties of the network, different metrics are also computed.

*Centrality indices* make implicit assumptions about the manner in which traffic flows through a network and are used to produce a ranking of the nodes according to their importance. *Degree centrality* emphasizes nodes with greater numbers of connections. *Betweenness* centrality highlights nodes that frequently occur in shortest paths among other nodes. The *Page rank centrality* reveals the importance of a node on the basis of its links to other central nodes [33, 34]. Centrality indices have been successfully used in epidemiology to identify individuals who can be pivotal in the spread of infections. As an example, the degree centrality was used to measure the probability of infection of a subject in terms of number of its exposures [35].

The *modularity* measure defines sub-networks or communities of nodes with higher level of linkage density or, in other words, highlights groups of nodes with shared attributes.

In this work we determined the modular structure of the network using an algorithm consisting of two phases iteratively repeated, in order to maximize the modularity within a community and to minimize it among different communities [32, 36].

### Study population and network implementation

In 2007, 148 GPs working within a Local Administrative Unit in Italy evaluated all patients (n = 23,996) aged 75 and over to classify them as to their status, ranging from not-frail to frail, without the use of a specific evaluation tool, but only using the criteria listed below.

GPs identified those elderly without any health or social needs as “not-frail” (NF). They also indicated those subjects characterized by lack of family support, lack of inclusion in the social network or having financial problems as “pre-frail with social needs” (PFSN). “Pre-frail with health needs” (PFHN), by comparison, were identified as elderly without social needs but with reduced ability to manage normal daily activities and also exhibiting non-managed or disabling diseases. Those patients affected either by health or social needs were defined “Frail” (FR). All data were collected through a detection module integrated into the GPs’ software (Millenium srl, Dedalus, Florence, Italy); for those few GPs not equipped with that software, a tool using EpiData (EpiData Association, Odense, Denmark) was implemented.

All patients were also classified using socio-demographic variables (age, gender, marital and family status), collected by GPs using the detection module.

Morbidity was evaluated through record-linkage between GPs’ data records and hospital admissions happened in year 2006, in order to assess the occurrence of previous illnesses that could be associated with the likelihood of being identified by GPs as frail or pre-frail.

All ICD9-CM (International classification of disease, ninth edition clinical modification) codes registered as the main and/or the secondary diagnosis of the hospital discharge records were grouped into 16 clinical categories using the H-CUP multilevel method, implemented by the U.S. Agency for Healthcare Research and Quality [37].

The linkage procedure was based on a de-identified numeric code originally assigned to each patient by the data provider (the only entity authorized to know the real identity of the patients), according to the Italian Legislative Decree 196/2003 on privacy.

Each final patient record, created though the linkage procedure, contained information about gender, age class, marital and family status, the presence or absence of co-morbidities (the clinical features) and the frailty status (FR) as identified by GPS. The disease variables were represented through dichotomous variables indicating the presence/absence of the disease.

The final data file was structured so that it could be represented using a graph. Creation of the social network layout of the graph and the actual analysis were carried out using Gephi [38], an open source software platform that allows interactive exploration and analysis of complex networks.

The data file loaded into Gephi generated an undirected graph: a node for every patient, a node for every characteristic and an edge to connect each patient node to all its characteristics nodes. The characteristic nodes are connected to each another whenever they refer to the same patient. Information about the graph’s layout and the relative algorithm are available in Franchini et al. [32].

Within the graph, the nodes and the labels sizes have been ranked using the centrality degree measure that highlights nodes with a higher number of connections in the network. The nodes’ colors revealed the communities identified by the modularity class measure.

The main centrality indexes and the network measures have been calculated in order to obtain metrics to be used in the result interpretation.

To determine the relative importance of the elderly characteristics within the net, the role of each one was evaluated by the comparison among the centrality scores.

The distributions of the centrality measures (Degree, Page rank, Betweenness) were divided into tenths (or deciles) and scored in a rank order from 1 to 10. A single score for any centrality measure was assigned to all the elderly features, with 10 the most influential. A total score for each characteristic was calculated as the sum of the individual scores.

Assuming a maximum potential score of 30 (3 centrality measures multiplied by the individual score of 10), the deviation from the maximum score was calculated for each feature.

Furthermore, to compare the centrality values of the different communities, the average deviation from the maximum score was derived from the ratio between the sum of the deviation of each modality and the number of modalities included in each community.

## Results

### Prevalence of frailty

Among the 23,996 elderly aged 75 years or older and evaluated by their GPs, frailty and pre-frailty prevalence were respectively 9.1 and 23.4 %: these estimates varied by age and were higher among women than men (Table 1). Women were overrepresented within the study population, in particular among frail, and were older than men in all groups.

**Table 1:** Overall network prevalence by frailty status

	Not Frails (NF) N = 16,188	Pre-frails with social needs (PFSN) N = 1098	Pre-frails with health needs (PFHN) N = 4521	Frails (FR) N = 2189
	N(%)			
Overall network prevalence^a^	67.5	4.6	18.8	9.1
By gender^a^				
Female	62.5	4.5	22.1	10.9
Male	74.9	4.7	14.0	6.4
Gender ratio (M/F)	0.8	0.7	0.4	0.4
By age^a^				
75–79	83.3	4.7	8.0	3.9
80–84	69.6	5.0	16.6	8.9
85–89	51.3	4.5	29.9	14.3
90+	31.4	2.8	46.2	19.6
Hospitalization within frailty status^b^	14.2	15.7	22.9	23.5
For multi-morbid profiles^b^	6.7	9.6	13.0	14.4
Circulatory d. + End-Metabolic d.	0.5	0.7	0.8	1.1
Circulatory d + Respiratory d.	0.3	0.3	0.6	0.5
Circulatory d + Digestive d.	0.4	0.4	0.3	0.2
Circulatory d + Injury-Poisoning	0.2	0.2	0.5	0.2
Circulatory d + Genito-Urinary d.	0.2	0.4	0.3	0.6
Circ. d. + End-Met. d. + Resp. d	0.1	0.5	0.2	0.3
Circulatory d. + Neoplasms	0.2	0.3	0.2	0.3
Neoplasms + Digestive d.	0.2	0.1	0.2	0.1
Neoplasms + Genito-Urinary.	0.2	0.3	0.1	0.0
Circulatory d + Sign-Symptoms	0.1	0.2	0.2	0.1
Circulatory d + Muscoloskeletal d.	0.1	0.5	0.1	0.3
Circulatory d. + Nervous d.	0.1	0.0	0.2	0.2
Circ. d. + Resp. d + Genito-Urinary	0.1	0.5	0.1	0.2
Circulatory d + Mental illness	0.1	0.2	0.1	0.3
Circ. d. + End-Met. d. + Gen-Urin. d	0.1	0.0	0.0	0.2
Other multi-morbid profiles	3.8	5.2	9.0	9.7

^a^Prevalence estimates referred to the overall study population

^b^Prevalence estimates specific for each frailty status

As shown in Table 1, the prevalence of elderly who had a previous hospitalization ranged between 14 and 24 %: frail’s showed the highest value and the greatest proportion of multi-morbid profiles (14.4 %). The most frequent multi-morbid profiles included circulatory, endo-metabolic, respiratory, digestive and genito-urinary diseases (Table 1).

### Results of the network analysis

In this study, the data set contained three types of nodes: the elderly (23,996 nodes), their characteristics (age, gender, marital status, family status, clinical features, split in 28 nodes as shown in Table 2) and the FR identified by GPs (split in 4 nodes). Our aim was to describe the ways in which groups of individuals, their characteristics and the GPs assessment are associable, through the analyses of the density of the edges which connect nodes to each another [39].

**Table 2:** Association among the socio-demographic and clinical features, the communities and the frailty status

Variables	Variables modalities	NF	PFSN	PFHN	FR	Not associated
Age class	75–79	Yellow				
	80–84	Yellow				
	85–89			Green		
	90+		Orange		Orange	
Gender	Male	Yellow				
	Female		Orange		Orange	
Marital status	Married	Yellow				
	Unmarried	Yellow				
	Widowed		Orange		Orange	
Family status	Living in a family without critical issue	Yellow				
	Elderly couples, two self-sufficient elderly	Yellow				
	Taking care of non-self-sufficient subjects	Yellow				
	Living alone		Orange		Orange	
	Living in an eldercare facility		Orange		Orange	
Clinical features	Injury and poisoning		Orange		Orange	
	Diseases of the blood					Blue
	Diseases of the circulatory system					Blue
	Diseases of the digestive system					Blue
	Endocrine, nutritional, met. and imm. disorders					Blue
	Diseases of the genitourinary system					Blue
	Infectious and parasitic diseases					Blue
	Mental illness					Blue
	Diseases of the musculosk. system and conn. tissue					Blue
	Neoplasm					Blue
	Diseases of the nervous system and sense organs					Blue
	Diseases of the respiratory system					Blue
	Diseases of the skin and subcutaneous tissue					Blue
	Symptoms, signs and ill-defined conditions					Blue

**Fig. 2:**
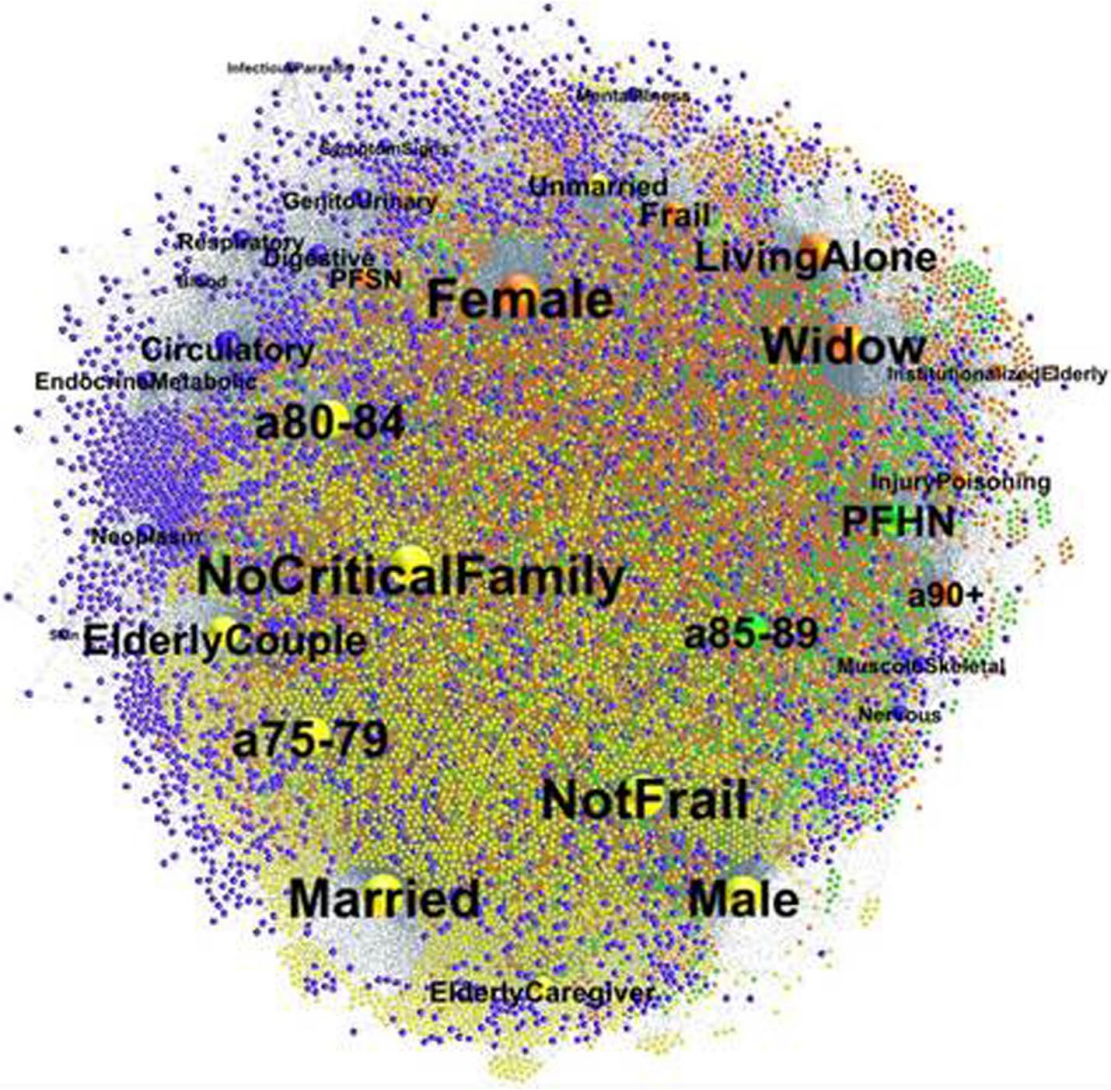
Network layout plotted by Gephi

Figure 2 showed the network layout (undirected graph) implemented by Gephi. The network contained 24,028 nodes and 128,360 edges: the size of each node was determined according to its centrality degree (Fig. 2). Colors were used to represent nodes belonging to a specific community in which they were more tightly connected. Modularity measure amounted to 0.197, within a range from −1 to 1.

This graph, represented in a two-dimensional projection, used labeled spheres to represent actors (individuals, their characteristics and frailty levels) and line segments between pairs of actors to represent the observation that a tie exists between the two. The node labels suggested the meaning of the variables and many of them are self-explanatory as ‘Male’ and ‘Female’ indicating patients gender. The labels ‘a75-79’, ‘a80-84’, ‘a85-89’and ‘a90+’ indicated the four age classes. Several clinical categories were represented in the graph using these labels: ‘Circulatory’, ‘EndocrineMetabolic’, ‘Digestive’, ‘Respiratory’, ‘Nervous’, etc.

The four FR identified by GPs were indicated with ‘NotFrail’, ‘PFSN’, ‘PFHN’ and ‘Frail’ respectively. Further, the three different marital status were represented by ‘Married’, ‘Widowed’ and ‘Unmarried’ nodes. Lastly, five separate family status were evident: ‘NoCriticalFamily’ that represented the condition of living in a family without critical issues, ‘ElderlyCouple’ that indicated an elderly but self-sufficient couple, ‘ElderlyCaregiver’ that indicated elderly taking care of non-self-sufficient relatives, ‘LivingAlone’ which indicated singles and ‘ElderlyInstitutionalized’ that represented living in an eldercare facility.

An immediate observation provided by the graph concerned the node positions. Smaller nodes were generally more peripheral to the network center e.g. Infectious and parasitic diseases, suggesting a lower level of interaction within the net. At the same time some nodes were closer to one another, suggesting a higher level of connection among some elderly features. So, for example, the endocrinemetabolic node placed near circulatory node suggested that there were many connections between the two disease categories in the network.

The node colors identified four communities including patients and their socio-demographic and clinical features (Table 2): yellow (10,312 patients), orange (7967 patients), green (2417 patients) and blue (3570 patients).

**Fig. 3:**
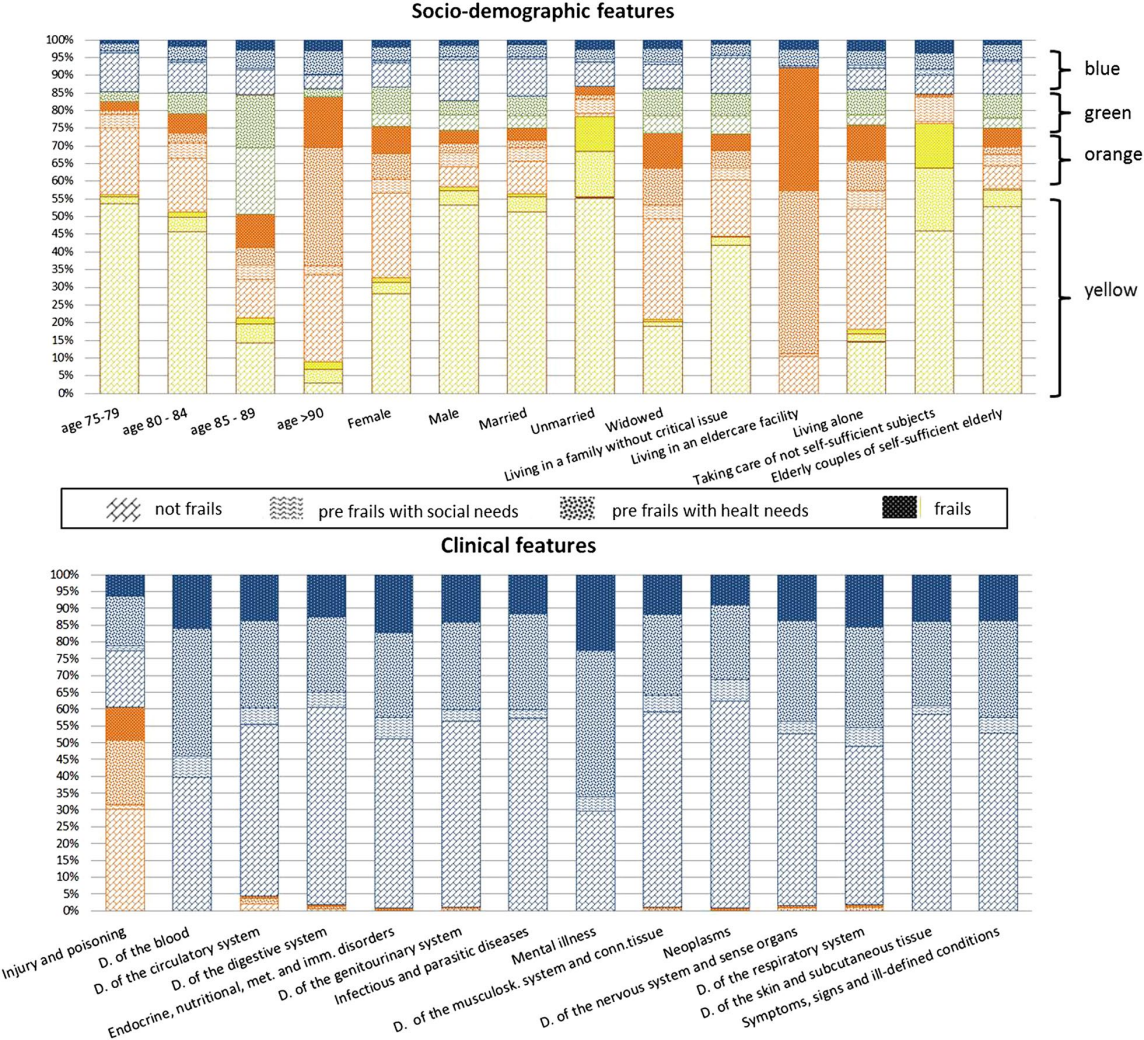
Percentage distribution of the socio-demographic and clinical features by frailty status and community

Figure 3 shows the percentage distribution of the socio-demographic and clinical features by FR and community.

In particular, the percentage of NF belonging to the yellow community totaled 57 %, the proportion of PFHN belonging to the green community was 33 % and within the orange community the percentage of PFSN and FR were 85 and 66 %, respectively (Fig. 3).

Moreover, the integration of socio-demographic and clinical features highlighted the association between the FR identified by GPs and the different profiles of the elderly (Table 2; Fig. 3).

Within the yellow community those identified as NF showed a higher density of linkage with male, age 75–84 years, married or unmarried marital status, living in a positive home environment (family without critical issues or elderly couples, with or without family ties, and composed of two self-sufficient elderly), or having a caregiving role (taking care of not self-sufficient subjects, aged of 75 years and over). Moreover those elderly did not show any association with previous experiences of hospitalization (in Fig. 3, the “clinical feature” graphical representation does not show any bar for the yellow community).

Older females, widowed, living alone or in eldercare facility, and being associated either with a PFSN or FR were included in the orange community (Table 2; Fig. 3). Those women were also strongly associated with a previous experience of hospitalization for injury and poisoning events. In the orange community those elderly who were hospitalized during the previous 12 months, showed a proportion of multi-morbidity profiles of 21 %, while 94 % of single ill profiles concerned injury and poisoning events.

Patients aged 85–89 years identified by GPs as PFHN, were mainly included in the green community (Table 2). This community did not show a clear association with previous hospitalizations.

On the contrary, the blue community was tightly linked with previous experience of hospitalization for all the clinical features, with exception of injury and poisoning events: all subjects of the blue community experienced at least one hospitalization during the last 12 months (Table 3). The distribution by FR within the blue community was more homogeneous and varied from 13 to 20 %, according to the frailty levels from NF to FR.

**Table 3:** Specific prevalence by frailty status within the blue community

	Not Frail (NF)	Pre-frail with social needs (PFSN)	Pre-frail with health needs (PFHN)	Frail (FR)
	N (%)			
Prevalence by frailty status	12.9	14.8	19.6	19.8
Gender ratio (M/F)	1.1	0.9	0.6	0.5
By hospitalization events:	12.9	14.8	19.6	19.8
For multi-morbid profiles:	6.4	9.4	12.3	13.7
Circulatory d. + End-Metabolic d.	0.5	0.7	0.8	1.1
Circulatory d + Respiratory d.	0.3	0.3	0.6	0.5
Circulatory d + Digestive d.	0.4	0.4	0.3	0.2
Circulatory d + Genito-Urinary d.	0.2	0.4	0.3	0.5
Circ. d. + End-Met. d. + Resp. d	0.1	0.5	0.2	0.3
Circulatory d. + Neoplasms	0.2	0.3	0.2	0.2
Neoplasms + Digestive d.	0.2	0.1	0.2	0.1
Neoplasms + Genito-Urinary.	0.2	0.3	0.1	0.0
Circulatory d + Sign-Symptoms	0.1	0.2	0.2	0.1
Circulatory d + Muscoloskeletal d.	0.1	0.5	0.1	0.3
Circulatory d. + Nervous d.	0.1	0.0	0.1	0.2
Circ. d. + Resp. d + Genito-Urinary	0.1	0.5	0.1	0.1
Circulatory d + Mental illness	0.1	0.2	0.1	0.3
Circ. d. + End-Met. d. + Gen-Urin. d	0.1	0.0	0.0	0.2
Other multi-morbid profiles	3.8	5.2	8.9	9.3

On average, the prevalence ratio between the proportion of multi-morbidity profiles within the blue community and the overall network proportion amounted to 95 %, with no relevant differences among FR.

The associations of diseases more frequently detected in the blue community were the same reported in Table 1, except for the association between circulatory diseases and injury-poisoning which only concerned the orange community.

The graph representation (Fig. 2) also supported these findings: while the pathologies included in the blue community are adjacent, the injury and poisoning class, belonging to the orange community, lied in a diametrically opposed position in two-dimensional projection.

### Network centrality measures

**Fig. 4:**
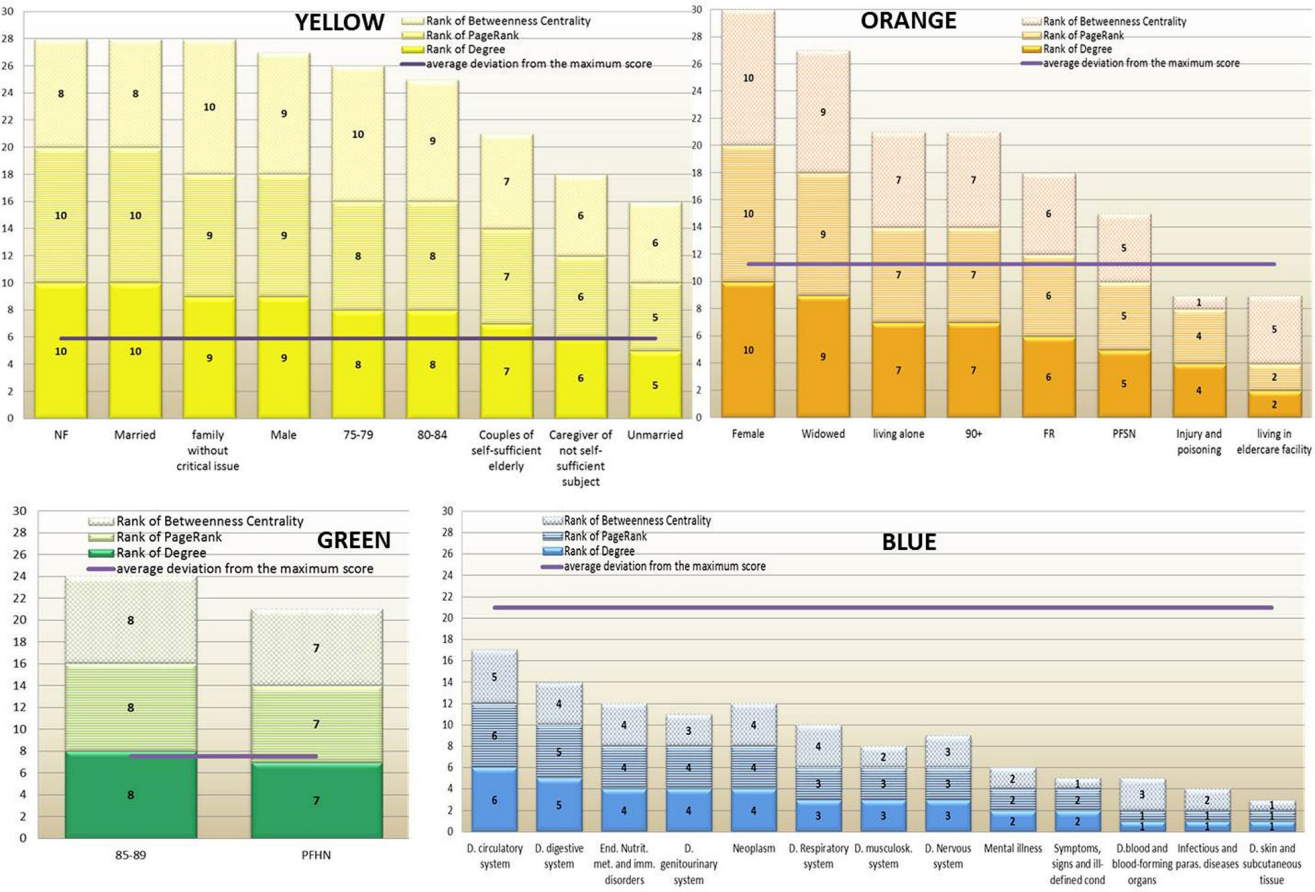
Distribution of the network centrality measures for each community

Figure 4 shows for each community the average deviation from the maximum potential score of 30 (horizontal lines) and the variable ranking distribution for each centrality measure (vertical bars).

According to the network centrality measures (Fig. 4), the more central nodes belonged to the yellow community, followed by the green and the orange: the average deviation from the maximum score was respectively 6, 8 and 11 points.

The blue community (average deviation from the maximum score is 21) had the lowest values of centrality measures and, as consequence, the lowest level of association either among its nodes or with the other communities. This result was clearly depicted in Fig. 2, where the blue nodes were mainly located in a more peripheral position, if compared to the nodes belonging to the other communities.

Focusing on the FR identified by GPs, the centrality measures showed a variability from single score of 5 concerning the node PFSN, to a score of 10 related to the node NF (Fig. 4).

In particular the node NF had the highest values in terms of Degrees and Page rank indexes; Betweenness value indicated that NF node also played a ‘broker’ role in the network (score 8).

The nodes PFHN and FR had a discrete level of association (Degree), even with high degree nodes (Page rank). The Betweeness value was also at an intermediate level of intensity (score 7).

Furthermore, the node PFSN belonging to the same community of FR node (orange community), had the lowest intensity of associations within the net (score 5), compared to the other FR.

## Discussion

This study involved some implicit and explicit assumptions:

1. Frailty is more than a simple accumulation of deficits, and effective management would be extremely costly if dependence on separate medical specialists was required to address each individually [40–42].
2. A chronic condition, by itself, is not the only deficit that increases frailty. Many non-chronic conditions, such as injuries, may also act as if they were chronic, especially if they reoccur over time, as in falls [14].
3. Disease-focused management in the elderly should be broadened to address person-oriented care over time to improve the likelihood that the healthcare system will remain still sustainable and more equitable [28, 43].
4. Following a whole-patient approach means having the ability to integrate the entire breadth of available patient data and to identify and gather critical information not currently available. This implies the capability of interpreting the available data from all the existing sources (e.g. administrative data flows), observing the elderly over time and being able to detect early signs of impairment, including use of the descriptive powers of the “narrative medicine” methods [44, 45].

It is widely accepted that GP is optimally positioned to meet those conditions [28] and the primary result of our analysis shows that the GP can best observe, identify and manage patient trajectories through the aging process. This early identification of patient clusters, to determine where a patient is likely headed, how far they have progressed and potentially how rapidly they are progressing will be essential to optimize care in a more personalized manner and provide more effective and cost-effective care long term.

The general observations of our study show that frailty prevalence estimated by GP assessment (9.1 %) is similar to those found in other studies [46, 47] that refer to more specific or comprehensive criteria. But more critically, although pre-frailty prevalence appears lower than those reported in other studies [7, 47, 48], GP evaluation reveals the true heterogeneity that can only be explained by the reality of separable patient trajectories, i.e. the communities that were identified through application of SNA [49].

SNA identified discrete features within the data that fit well with the GP’s assessment of frailty levels. GPs tend to identify females aged 90 years and older, widowed, living alone or in eldercare facility, as frail elderly or pre-frails with social needs. This evidence is in line with those reporting a high level of frailty among older women [20]. At the same time this result emphasizes the association between early signs of frailty and its social determinants, such as the lack of family support, the lack of inclusion in the social network or existing financial problems: this emphasizes the GPs’ attitude towards both patients’ experiences of illness and of life [28, 42].

Furthermore, from a clinical point of view, the evidence of association between frailty and previous experiences of hospitalization for injury events may result from the increased levels of inflammatory mediators which are strongly related to the frailty syndrome. The cytokine IL-6 is a major factor modulating muscle mass and ultimately causing sarcopenia as well as reduction in bone density [50]. Moreover, poly-pharmacy showing a prevalence rate from 4 to 42 % among the elderly in the outpatient services [51], is widely reported as risk factors of falls [52] and as we have reported, also using SNA [32].

The association between older age, female gender and injury/poisoning is also consistent with existing literature. Declining health, multi-morbidities, cognitive dysfunction and depression may contribute to accidental and deliberate poisoning as well as age-related physiological changes and poly-pharmacy can affect the pharmacokinetic and pharmacodynamic profiles of drugs, making elderly more susceptible to toxicity [52, 53]. Cassidy et al. [52] reported a female/male ratio of 1.6 within a sample of elderly who experienced poisoning. These events occurred mainly in a domestic setting (87 %) while nursing home represents the second place of occurrence (4.5 %).

Our results show that from the GPs’ perspective the pre-frailty condition due to health needs mainly concerns patients aged 85–89 years, independently either from their gender or from the experience of previous hospitalizations during the last 12 months. This result seems to confirm the abilities of GPs in detecting early signs of impairment, relying more on the relational continuity with their patients than single adverse health events such as hospitalization. This is exactly in line with the early identification of a patient trajectory and the potential to intervene to better manage their healthcare and costs.

Further support comes from the analysis of those elderly belonging to the blue community. Firstly, even though subjects having past experiences of hospitalization during the previous 12 months belong to a single community, the proportion of frail and pre-frail with health needs is higher (more than 19.0 %) if compared to the lowest levels of frailty (less than 15 %). Secondly, within the blue community the prevalence of multi-morbidity profiles varies according to the frailty levels from not frail (6.4 %) to frail (13.7 %). These results indicate that in identifying frailty severity, GPs consider the accumulation of deficit paradigm, referring to the multi-morbidity condition.

The validity of the GP’s approach in diagnosing frailty in the elderly is also supported by the evidence that younger males, without previous hospitalizations, and living in a positive home environment or acting as caregiver, are mainly identified as not frail.

Moreover, this study is based on several key strengths. In particular its large patient population and the significant number of GPs involved (148). Furthermore, participating GPs represent different perspectives in terms of work site (rural/urban), practice experience and gender.

Operationally, this study involved lower costs and required less time than prospective survey, due to the availability of administrative data and electronic record use by the GPs.

In summary, our findings provide evidence that GPs are able to perform low cost, high value population-based evaluation. Their approach to patients, combined with the information available derived from their daily practice, can be used to establish biomarkers/diagnostics for early identification and stratification of patients to improve their quality of care and potentially optimize costs.

As a consequence integrating GPs’ routinely collect data into a health patient record, combined with other electronic health documents already available, could provide a comprehensive picture of the health state of patients even beyond attempting to enhance the diagnosis of patient “state”.

This approach could provide a valuable experience for implementing effective health promotion and disease prevention programs that will have major implications for a country’s future burden of disease.

This is particularly relevant in many developing countries where older population are growing more rapidly than are those of industrialized nations.

In China the transition of the population over age sixty to double will take only thirty-four years, in Venezuela, only twenty-two years, while in European countries it took more than 100 years [54].

In developing countries there is still the opportunity and the challenge to improve healthcare systems to face the rapid shifting of the predominant illness profile, from acute to the chronic diseases.

Finally, we believe that this study further demonstrates that network analysis is an extremely flexible research tool and a rich theoretical paradigm. In the healthcare field this approach can provide a useful framework, particularly suited to explore the reality of complex phenomena that pervades healthcare, thus reducing the gap between data and knowledge.

There are numerous opportunities in public health practice to use a network approach for developing and implementing health intervention, and further studies should be developed, particularly referring to the stochastic and longitudinal network analysis models.

## Abbreviations

GPs: general practitioners
SNA: social network analysis
NF: not-frail
PFSN: pre-frail with social needs
PFHN: pre-frail with health needs
FR: frail
